# Fabrication of mAb G250-SPIO Molecular Magnetic Resonance Imaging Nanoprobe for the Specific Detection of Renal Cell Carcinoma *In Vitro*

**DOI:** 10.1371/journal.pone.0101898

**Published:** 2014-07-07

**Authors:** Cailuan Lu, Jingjing Li, Kai Xu, Chun Yang, Jiali Wang, Cuiping Han, Xiaohua Liu

**Affiliations:** 1 Department of Radiology, Affiliated Hospital of Xuzhou Medical College, Xuzhou, Jiangsu Province, PR China; 2 School of Medical Imaging, Xuzhou Medical College, Xuzhou, Jiangsu Province, PR China; Brandeis University, United States of America

## Abstract

Molecular magnetic resonance imaging (mMRI) has been paid more and more attention for early diagnosis of cancer. A sensitive and specific mMRI probe plays the most important role in this technique. In this study, superparamagnetic iron oxide (SPIO) nanoparticles and mAb G250 were conjugated as mMRI probe for the detection of clear cell renal cell carcinoma (ccRCC) using 3.0-Tesla MRI *in vitro*. mAb G250 could specifically recognize carbonic anhydrase IX (CAIX) antigen overexpressed in ccRCC and the SPIO nanoparticles as MRI contrast agent presented excellent MRI response and good biocompatibility. The successful assembly of this nanoprobe was confirmed by UV-vis spectrum, FT-IR spectroscopy and DLS analysis. *In vitro* MRI study on ccRCC cells and control cells indicated that our fabricated mAb G250-SPIO nanoprobe could be used in the specific labeling of clear cell renal carcinoma cells successfully.

## Introduction

Renal cell carcinoma (RCC), which arises from renal tubular epithelial cells [1]–[2], accounts for approximately 90% of all renal malignancies [3]. It was estimated to be diagnosed in over 60,000 individuals in the United States in 2011 [4]. The main subtype of RCC is clear cell RCC (ccRCC, approximately 70%), followed by papillary RCC (7% to 15%), chromophobe RCC (3%–5%), and a variety of other rare histologic subtypes [5]–[7]. Due to its high degree of malignancy and difficulty in early detection, the mortality of RCC is high. Thus, looking for an approach to realize early diagnosis and therapy of RCC is essential for patient survival and quality of life. Although medical imaging diagnosis technology has been rapidly developing in recent years, few reports have emerged for its application in the early diagnosis of RCC.

Carbonic anhydrates IX (CAIX) antigen is a cytosolic transmembrane glycoprotein and can be recognized by the IgG monoclonal antibody (mAb) Grawitz (G250) [8], [9], which was first discovered by Oosterwijk in 1986 [10]. CAIX catalyzes the reaction: CO_2_+H_2_O↔HCO_3_^−^+H^+^, which is vital to the regulation of proton flux in cells and pH values [11]. It is highly expressed in 97% to 98% ccRCC in both primary and metastatic disease but is lowly expressed or absent in papillary RCC, chromophobe RCC and oncocytoma and absent in normal kidney tissues [9], [12], [13]. This makes CAIX antigen an ideal biomarker for the diagnosis of ccRCC. Radionuclide imaging using mAb G250 for specific targeting of the CAIX antigen in ccRCC has been reported [14]–[18]. However, the low resolution for diagnosis and its damage to the normal tissue hampered its further applications.

Magnetic resonance imaging (MRI), which provides superior resolution, unlimited penetration into tissue and no ionizing radiation, is one of the best strategies used in clinic to diagnose cancer [19]. In order to improve the visibility of internal body structures, various MRI contrast agents (CAs) have been introduced by shorting the relaxation parameters (T1 and T2) of water. Chelated gadolinium compounds such as Gd-DTPA and Gd-DOTA are the mostly used positive CAs [20]. However, the low relaxivity, short blood circulation time and low specificity limit their further applications. Compared to Gd-based contrast agents, superparamagnetic iron oxide (SPIO) nanoparticles as negative CAs have been widely used in MRI for both vascular imaging and tumor imaging. The main advantages of SPIO nanoparticles as MRI CA include their high signal strength, longer lasting contrast enhancement and relatively low cytotoxicity [21], [22]. Additionally, the iron released from degrading SPIO nanoparticles can also be metabolized by body, reducing the possibility for long-term cytotoxicity [21]. More importantly, the easy functionalization of SPIO nanoparticles shows their potential in molecular magnetic resonance imaging (mMRI). Conjugation of SPIO nanoparticles with mAb offers the possibility of mMRI of tumor biomarkers. In 2005, Toma A et al [23] realized targeted SPIO imaging for rectal carcinoma using monoclonal antibody-7. Later in 2012, Koyama T et al. [24] applied mAb-conjugated dextran-coated SPIO nanoparticles (CMDM) for the diagnosis of tumor-bearing mice *in vivo*. The purpose of our study was to develop mAb G250-conjugated SPIO nanoparticles as an mMRI probe to specifically and efficiently target ccRCC for early diagnosis. To the best of our knowledge, this is the first report of ccRCC detection by mMRI based on mAb G250-conjugated SPIO molecular nanoprobe.

## Materials and Methods

### Chemical and Materials

Superparamagnetic iron oxide (SPIO) nanoparticles functionalized by amino group were kindly provided by Jun-Jie Zhu’s group (State Key Laboratory of Analytical Chemistry for Life Science of Nanjing University). 1-ethyl-3-(3-dimethylaminopropyl) carbodiimide hydrochloride (EDC⋅HCl) and N-hydroxysuccinimide (NHS) were purchased from Sigma-Aldrich (USA). Bovine serum albumin (BSA) was obtained from Solarbio (China). Tween-20 was bought from Biosharp (Korea) and gelatin was purchased from Tianjin Kemiou Chemical Reagent Company. CCK-8 kit was purchased from Dojindo Laboratories. Specific mAb G250 was purchased from Sino Biological Inc. Nonspecific IgG mAb was purchased from Biogot Biotechnology CO, Ltd. All aqueous solutions were prepared in ultrapure water (≥18 MΩ, Milli-Q, Millipore).

### Apparatus and Characterization

UV−vis spectra were recorded on a UV-3600 spectrophotometer (Shimadzu, Kyoto, Japan), and FT-IR spectra were carried out on a Nicolet 400 Fourier transform infrared (FT-IR) spectrometer (Madison, WI). The size and morphology of SPIO nanoparticles were observed by transmission electron microscopic (TEM) (TECNAI G2, USA) and dynamic light scattering (DLS) (NiComp380ZLS, USA). Samples were prepared by placing a drop of SPIO nanoparticles suspension onto a copper grid and air-dried. Then, the samples were directly examined under the TEM. MRI scanning was performed on 3.0 T human magnetic resonance scanner (Signa, USA).

### Cells and Cell Culture

The ccRCC (786-0) cell line with CAIX antigen overexpressed was obtained from the Key Laboratory for Cancer Biology Treatment of Xuzhou Medical College as a gift. EA.hy926 normal human umbilical vein endothelial cell line (HUVEC) that is absent of CAIX antigen expression and human lung fibroblast HLF-1 cells were kindly provided by Teng Fei (Laboratory of Medical Imaging of Xuzhou Medical College). The original source of these three kinds of cell lines was from the same commercial source, the Cell Bank of the Chinese Academy of Sciences (Shanghai, China). NIH-3T3 mouse fibroblast cells were also purchased from it. The 786-0 renal carcinoma cells were cultured in a 10% FBS-containing RPMI 1640 medium (Gibco, Grand Island, NY) supplemented with penicillin (100 µg/mL), and streptomycin (100 µg/mL). NIH-3T3 and HUVEC cells were propagated in 10% FBS-containing DMEM medium (Gibco, Grand Island, NY) supplemented with penicillin (100 µg/mL), and streptomycin (100 µg/mL). HLF-1 cells were cultured in Ham’s F12 nutrient medium (Gibco, Grand Island, NY) supplemented with 10% FBS, penicillin (100 µg/mL), and streptomycin (100 µg/mL). All cells were grown in a humidified incubator (Thermo, USA) at 37°C under 5% CO_2_ atmosphere.

### MRI behavior test of SPIO nanoparticles

SPIO nanoparticles (2.32 mg) and sodium citrate (10 mg) were dissolved in 10 mL of double distilled water by vortexing and sonication at room temperature (the final concentration was 1000 µmol/L). Subsequently, a series of concentrations containing 0, 20, 40, 60, 80, and 100 µmol/L SPIO nanoparticles were diluted accordingly. Then, the MRI behavior of SPIO nanoparticles with various concentrations were evaluated by 3.0 T human magnetic resonance scanner (Signa, USA). The following parameters were adopted in data acquisition: 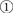 T2 weighted images: echo time (TE) = 90.7 ms, repetition time (TR) = 4500 ms, field of view (FOV) = 14 cm×14 cm, matrix = 384×224, slice thickness = 2 mm; 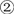 T2-map images: TE = 40 ms, TR = 3000 ms, FOV = 14 cm×14 cm, matrix = 384×224, slice thickness = 2 mm. Quantitative T2 relaxation maps were reconstructed from datasets using function software at a workstation (ADW 4.2). The signal intensity of the samples was measured, and the T2 values were calculated accordingly.

### Optimization of blocking agent

To reduce the non-specific binding of SPIO nanoparticles, the blocking effect of SPIO nanoparticles with Tween-20 and BSA was evaluated. 100 µg/mL SPIO nanoparticles were suspended in 500 µL of PBS containing 1% Tween 20 or 1% BSA for 1 h at room temperature. After washing with PBS for 3 times, the Tween 20 or BSA-blocked SPIO nanoparticles were dispersed in 500 µL PBS and stored at 4°C before use. Pure SPIO nanoparticles were used as control.

A total number of 3×10^4^ 786-0 renal carcinoma cells was seeded on each well of a 12-well plate. After 24 h, cells were incubated with 100 µg/mL of SPIO nanoparticles, 100 µg/mL of Tween-blocked SPIO nanoparticles and 100 µg/mL of BSA-blocked SPIO nanoparticles for 1 h at 4°C, respectively. After discarding the nanoparticles, cells were washed with PBS, lysed with trypsin and then harvested by centrifugation at 3000 rpm for 10 min. The obtained cells were resuspended in 500 µL PBS for MRI scanning. Cells incubated with culture medium only were used as control.

### Conjugation and characterization of mAb G250 with SPIO nanoparticles

The SPIO nanoparticles were conjugated with mAb G250 to obtain the specific mMRI nanoprobe through a covalent link between the carboxyl group (-COOH) of the antibody and the amino group (-NH_2_) on the surface of SPIO nanoparticles. Briefly, 10 µL of 1.56 µg/µL G250 antibody solution was mixed with 40 µL EDC⋅HCl (10 mg dissolved in 1 mL of PBS solution, pH 7.4) for 15 min at room temperature to activate carboxyl group. Then, 40 µL NHS (5 mg dispersed in 1 mL of PBS solution, pH 7.4) was added into the reaction system for another 15 min. Later, 500 µL of 40 µg/mL SPIO nanoparticles was added immediately, mixed and incubated for 2 h at room temperature. The conjugates were washed with PBS for 3 times to remove excess antibody and reagents and suspended in 500 µL of PBS containing 1% BSA for 1 h to block nonspecific binding sites. After washing with PBS for 3 times, the BSA-blocked nanoprobe was dispersed in 500 µL PBS and stored at 4°C before use. The nonspecific IgG antibody conjugated nanoparticles were prepared via the same procedure. Additionally, BSA-blocked pure SPIO nanoparticles without antibody conjugation were prepared and used as negative control. 500 µL of 40 µg/mL SPIO nanoparticles were incubated with 500 µL of 1% BSA for 1 h and after three washes with PBS were kept in 500 µL PBS.

For confirmation of successful conjugation, 500 µL of 40 µg/mL G250-mAb-SPIO nanoprobe without BSA blocking, 500 µL of 40 µg/mL untreated SPIO nanoparticles, and 500 µL of G250 antibody solution (10 µL of 1.56 µg/µL dispersed in 490 µL of PBS solution) were characterized by UV-vis spectrum and FT-IR spectroscopy, respectively. The hydrodynamic diameter of 40 µg/mL SPIO nanoparticles, G250-mAb-SPIO nanoprobe and BSA-blocked G250-mAb-SPIO nanoprobe were also analyzed by DLS to confirm the successful couple.

### Stability test of BSA-blocked SPIO nanoparticles

20 µg SPIO nanoparticles were incubated with 500 µL 1% BSA for 1 h and after three washes with PBS, they were dispersed in 500 µL cell culture medium for 1 h, 5 h, and 24 h as well as in 500 µL PBS for 1 h as control. After washed with PBS at different time point, the nanoparticles were suspended in 400 µL 30% gelatin solution for MRI scanning.

### Cell cytotoxicity test

Cytotoxicity testing of SPIO nanoparticles was performed by monitoring the mitochondrial reduction of CCK-8 kit as follows. The 786-0 renal carcinoma cells and normal human umbilical vein endothelial cells were seeded on 96 well plates at a cell density of 5×10^3^ cells/well. After culture in a 5% CO_2_ incubator at 37°C for 24 h, different concentrations of SPIO nanoparticles (10, 20, 40, 60, 80 and 100 µg/mL) in a serum-free medium were added to the plate and co-cultured for 12 h. The medium containing SPIO nanoparticles were discarded. The cells were rinsed twice with PBS, and cultured with fresh medium and 10 µL of the kit reagent per well. After incubation for 4 h, the absorbance for each sample was determined by microplate reader (Multiskon MK3, USA) at 450 nm. Untreated cells were taken as a control with 100% viability, and a well with fresh medium and 10 µL kit reagent was used as blank to calibrate the spectrophotometer to zero absorbance. The relative cell viability (%) compared to control cells was calculated by (Asample/Acontrol) ×100%. All experiments were repeated in triplicate.

### *In vitro* MR imaging

The 786-0 renal carcinoma cells were seeded in 12-well plates at a density of 3×10^4^ cells/well and cultured for 2 days in a humidified incubator at 37°C under 5% CO_2_ atmosphere. Then, the culture media was removed, and the cells were washed with PBS twice. Afterward, 40 µg/mL of G250-mAb-SPIO nanoprobes, 40 µg/mL of IgG-SPIO nanoparticles, 40 µg/mL of BSA-blocked SPIO nanoparticles or culture medium were added to each well, followed by an 1 h incubation at 4°C. After discarding nanoprobe, nanoparticles and culture medium, the cells were washed and lysed with trypsin. The cells were harvested by centrifugation at 3000 rpm for 10 min and resuspended in 400 µL 30% gelatin solution and kept at 4°C for MRI scanning. NIH-3T3 cells, HLF-1 cells and HUVEC cells were treated with 40 µg/mL of G250-mAb-SPIO nanoprobes, 40 µg/mL of BSA-blocked SPIO nanoparticles and culture medium with the same procedure as control.

*In vitro* MR imaging test was performed using a 3.0 T human magnetic resonance scanner and the parameters were set the same as that with SPIO nanoparticles mentioned previously.

## Results and Discussions

### Characterization and MRI behavior of SPIO nanoparticles

**Figure 1:**
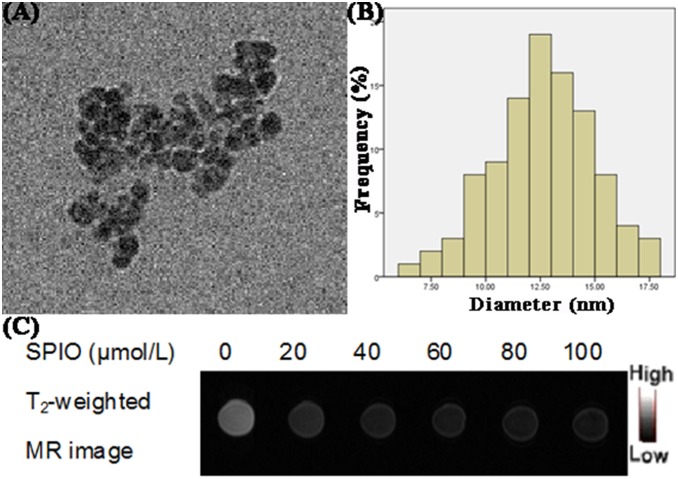
Characterization of SPIO nanoparticles. (A) TEM image of SPIO nanoparticles. (B) Distribution of SPIO nanoparticle size. (C) T2-weighted MR image of SPIO nanoparticles with different concentrations at 3.0 T. With increasing SPIO concentration, the MR image became darker.

The SPIO nanoparticles used in this study were synthesized according to a well-established coprecipitation approach and their size and morphology were characterized by transmission electron microscopy (TEM) and dynamic light scattering (DLS). As shown in Figure 1, the nanoparticles had a diameter of approximately 13 nm, which was smaller than the hydrodynamic diamer (33.6±5.1 nm) determined by DLS. Since the hydrodynmaic diameter is the sum of the core size and the thickness of adsorbed molecules layer, the hydrodynamic diameter is larger than the diameter estimated from a visual examination of TEM [25]. It was reported that nanoparticles with the size between 10 nm and 100 nm are the best fit for biomedical applications, because they have the longest circulation time, and their reduced surface area minimizes space for adsorption of reticuloendothelial system (RES) proteins [26].

As a negative CA, the MRI contrast effect of SPIO nanoparticles is important for their further biomedical applications. To evaluate their MRI contrast effect, a study was performed with SPIO nanoparticles of elevated concentrations at 3.0 T human magnetic resonance scanner. For T2 weighting, the higher the concentration of negative CA, the lower the signal intensity observed. As shown in Figure 1C, the signal intensity decreased with an increase in SPIO concentrations. The values of SPIO nanoparticles from 0 to 100 µmol/L were 497.67, 64.02, 53.09, 50.33, 46.07, 45.47 ms, respectively, which were determined by function software at a workstation (ADW 4.2). These results of MRI scanning showed that SPIO nanoparticles can noticeably reduce the T2 relaxation time and can be used as MRI contrast agent in following study.

### Optimization of blocking agent

**Figure 2:**
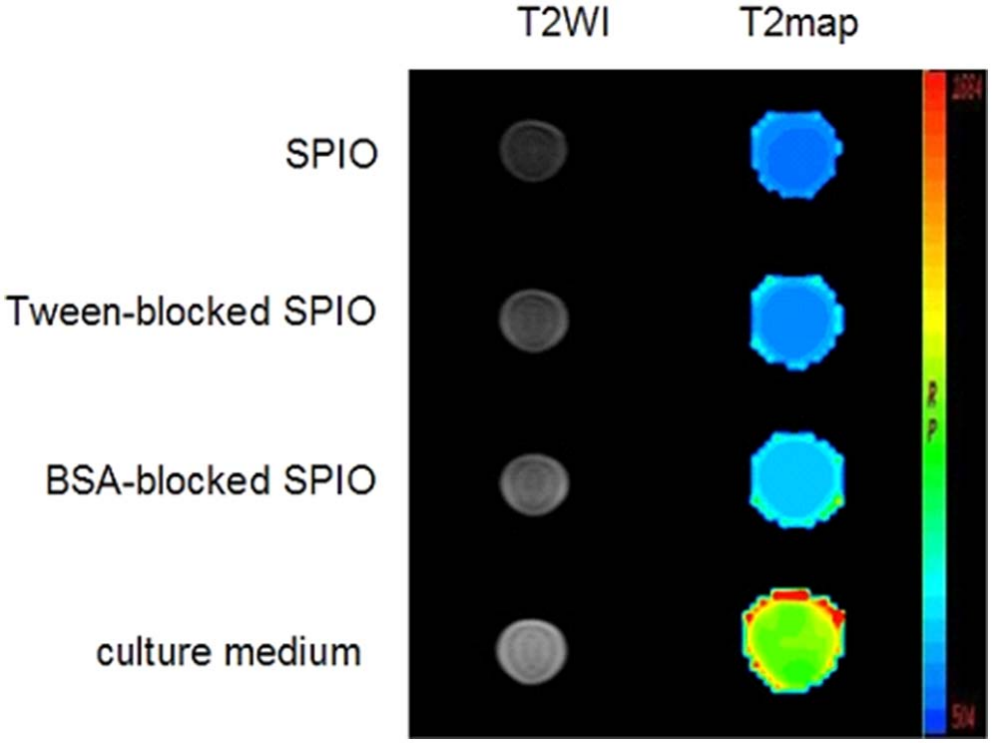
T2-weighted and T2map MR images of 786-0 renal carcinoma cells treated with SPIO nanoparticles, Tween 20-blocked SPIO nanoparticles, BSA-blocked SPIO nanoparticles, or culture medium.

To reduce the non-specific binding of SPIO nanoparticles, the blocking effect of SPIO nanoparticles with Tween-20 and BSA was evaluated. SPIO nanoparticles, Tween-20 and BSA-blocked SPIO nanoparticles were incubated with 786-0 renal carcinoma cells for 1 h at 4°C, respectively. After remove of nanoparticles, 786-0 cells were washed and harvested for MR scanning (Figure 2). The T2 relaxation times of each group were calculated by function software at a workstation (ADW 4.2) and the values for SPIO nanoparticles, Tween 20-blocked SPIO nanoparticles, BSA-blocked SPIO nanoparticles and culture medium were 87.72±12.34, 106.90±11.84, 145.75±23.13, 275.69±33.59 ms, respectively. Quantitative analysis of samples showed that BSA-blocked SPIO nanoparticles displayed the weakest non-specific adsorption to 786-0 renal carcinoma cells.

### Cell cytotoxicity

**Figure 3:**
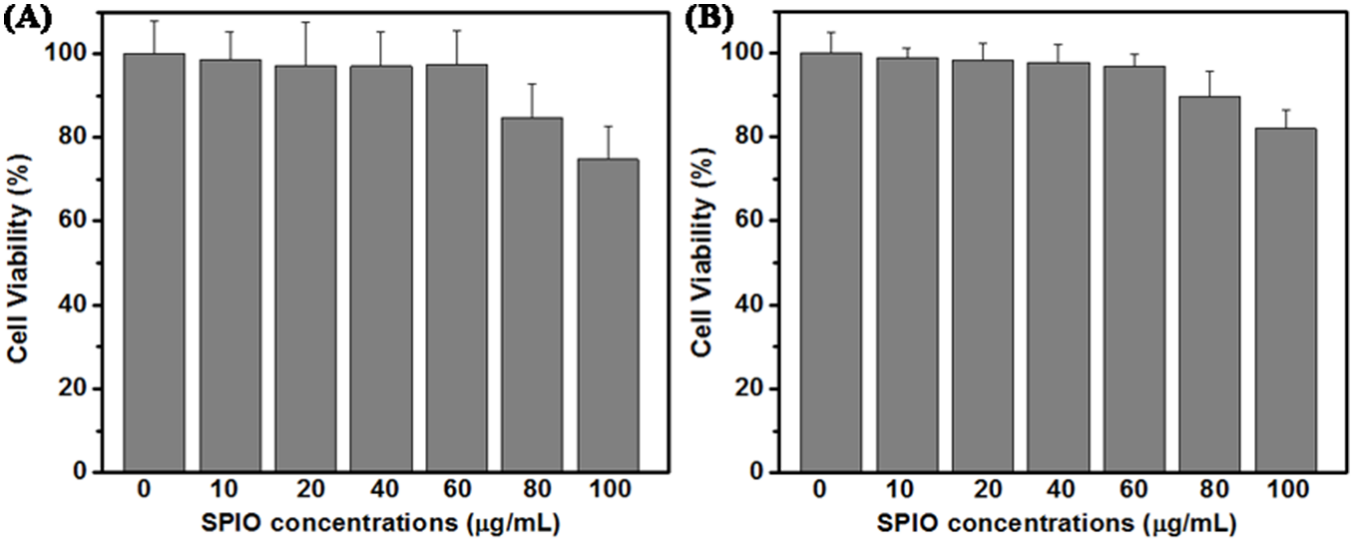
Cell viability of 786-0 renal carcinoma cells (A) and normal human umbilical vein endothelial cells (B) after exposure to various concentrations of SPIO nanoparticles, as determined by a CCK-8 assay.

The cytotoxicity of nanoparticles commonly arises from the production of excess reactive oxygen species (ROS), including free radicals such as the superoxide anion, hydroxyl radicals, and the non-radical hydrogen peroxide [27]–[29]. Cells are damaged if exposed to high levels of ROS [30]. Thus, it is vital to assess the safety of nanoparticles before clinical applications. The cellular biocompatibility of SPIO nanoparticles in our study was evaluated by a cell counting kit-8 (CCK-8) approach. The CCK-8 approach was based on the conversion of water-soluble tetrazolium salt, WST-8[2-(2-methoxy-4-nitrophenyl)-3-(4-nitrophenyl)-5-(2,4-disulfophenyl)-2H- tetrazolium, monosodium salt] to a water-soluble formazan dye upon reduction in the presence of an electron carrier by dehydrogenases [31], [32]. The SPIO nanoparticles with six different concentrations, ranging from 10 to 100 µg/mL, were incubated with 786-0 renal carcinoma cells and normal human umbilical vein endothelial cells for 12 h, respectively. As shown in Figure 3, the SPIO nanoparticles displayed good biocompatibility, and no significant cytotoxicity both on 786-0 renal carcinoma cells or normal human umbilical vein endothelial cells was observed even under a high concentration of 60 µg/mL. The cell survival rates of all groups were higher than 90% at the dose of 60 µg/mL (Figure 3). The concentration of 40 µg/mL SPIO nanoparticles was chosen for the following experiments.

### Characterization of specific mMRI probe

**Figure 4:**
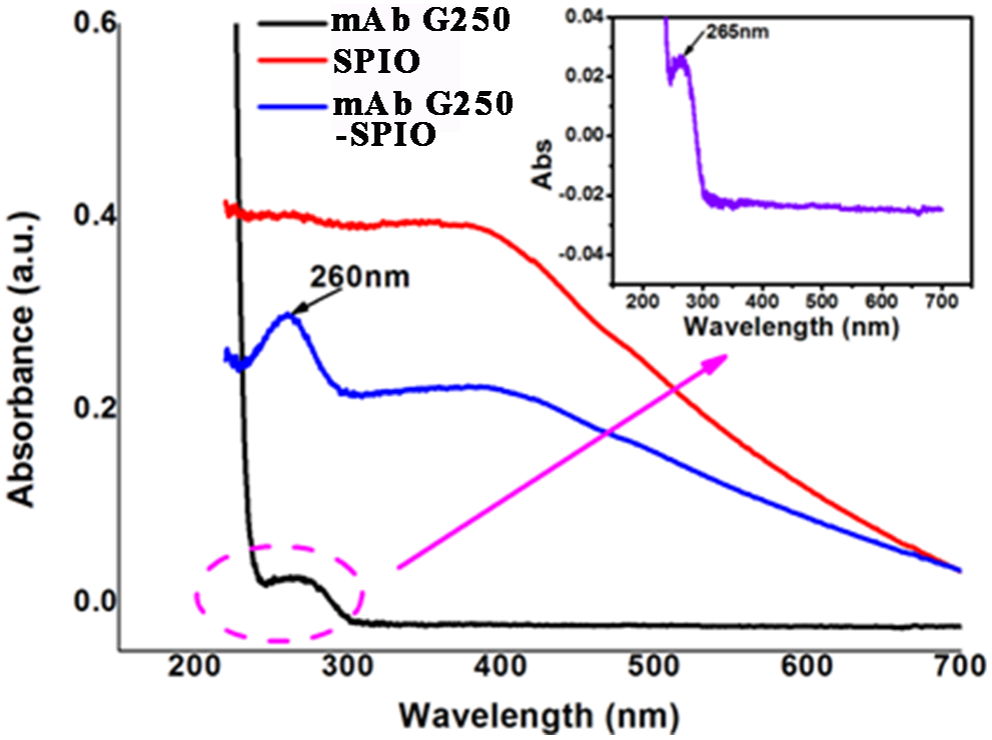
UV-vis absorption spectra of mAb G250-SPIO nanoprobes (blue line), SPIO nanoparticles (red line), and mAb G250 (black line). The emergence of absorption peak of mAb G250 in the mAb G250-SPIO nanoprobe indicates the successful fabrication of specific MR imaging nanoprobe.

**Figure 5:**
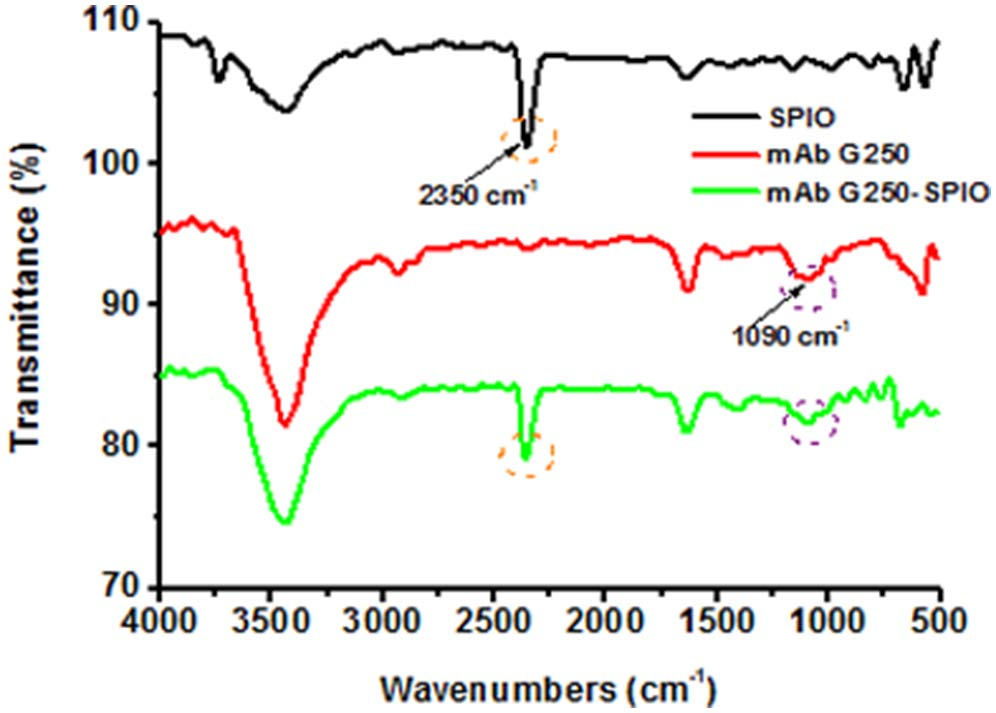
FT-IR spectra of mAb G250-SPIO nanoprobes (green line), SPIO nanoparticles (black line), and mAb G250 (red line).

G250-mAb-SPIO mMRI nanoprobe was prepared by covalent coupling method with the help of EDC⋅HCl and NHS. Such conjugation was characterized by UV-vis spectrum, FT-IR spectroscopy and DLS analysis. As shown in Figure 4 and Figure 5, both UV-vis spectrum and FT-IR spectroscopy showed the characteristic absorption peak of G250-mAb from G250-mAb-SPIO nanoprobe, which confirmed that G250-mAb had been successfully assembled to the surface of SPIO nanoparticles. In Figure 4, G250-mAb showed the characteristic peak at 265 nm, and G250-mAb-SPIO nanoprobe showed similar peak at 260 nm, indicating that G250-mAb were assembled onto the nanoparticle surface. Meanwhile, about 5 nm blue-shift in the UV-vis absorption peak of the antibody was observed after the conjugation. This might be ascribed to the near field coupling between adjacent particles [33]. FT-IR spectrum also indicated the successful preparation of G250-mAb-SPIO nanoprobe. The characteristic absorption peak at 1090 cm^−1^ for C-O of G250-mAb and 2350 cm^−1^ of SPIO nanoparticles were both preserved in G250-mAb-SPIO nanoprobe (Figure 5).

The successful conjugation was also confirmed by DLS analysis. Compared with SPIO, the hydrodynamic size of G250-mAb-SPIO nanoprobe was increased from (33.6±5.1) nm to (44.5±4.8) nm. With BSA blocking, the hydrodynamic diameter was further increased to (50.0±6.1) nm. The size increase of nanoprobe could be attributed to the conjugation of G250-mAb and BSA. The Zeta-potential of BSA-blocked G250-mAb-SPIO nanoprobe was −27.02±0.93 mV, which might come from the negative Zeta-potential of BSA.

### Stability test of BSA-blocked SPIO nanoparticles

**Figure 6:**
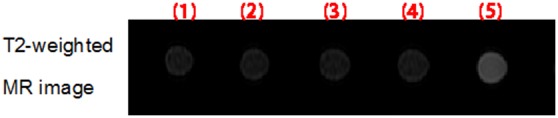
MRI stability test of BSA-blocked SPIO nanoparticles in cell culture medium for various hours. (1) BSA-blocked SPIO nanoparticles were dispersed in PBS for 1 h. (2)–(4) BSA-blocked SPIO nanoparticles were dispersed in cell culture medium for 1 h, 5 h, and 24 h. (5) pure 30% gelatin solution.

For the specific detection of 786-0 cells *in vitro*, the MRI stability of nanoprobe in the biological environment is important. Thus, BSA-blocked SPIO nanoparticles were incubated with cell culture medium for different time and their MRI behavior was evaluated accordingly. As shown in Figure 6, the T2 signal intensity of nanoparticles treated with cell culture medium for 1 h, 5 h, and 24 h were 396.86±±14.02, 397.40±18.19, 404.85±11.62, respectively. There was no significant difference from that in PBS solution (412.11±13.76) even after 24 h incubation with cell culture medium. Therefore, BSA-blocked SPIO nanoparticles were stable enough as mMRI nanoprobe for the detection of 786-0 renal carcinoma cells *in vitro*.

### *In vitro* MR imaging

**Figure 7:**
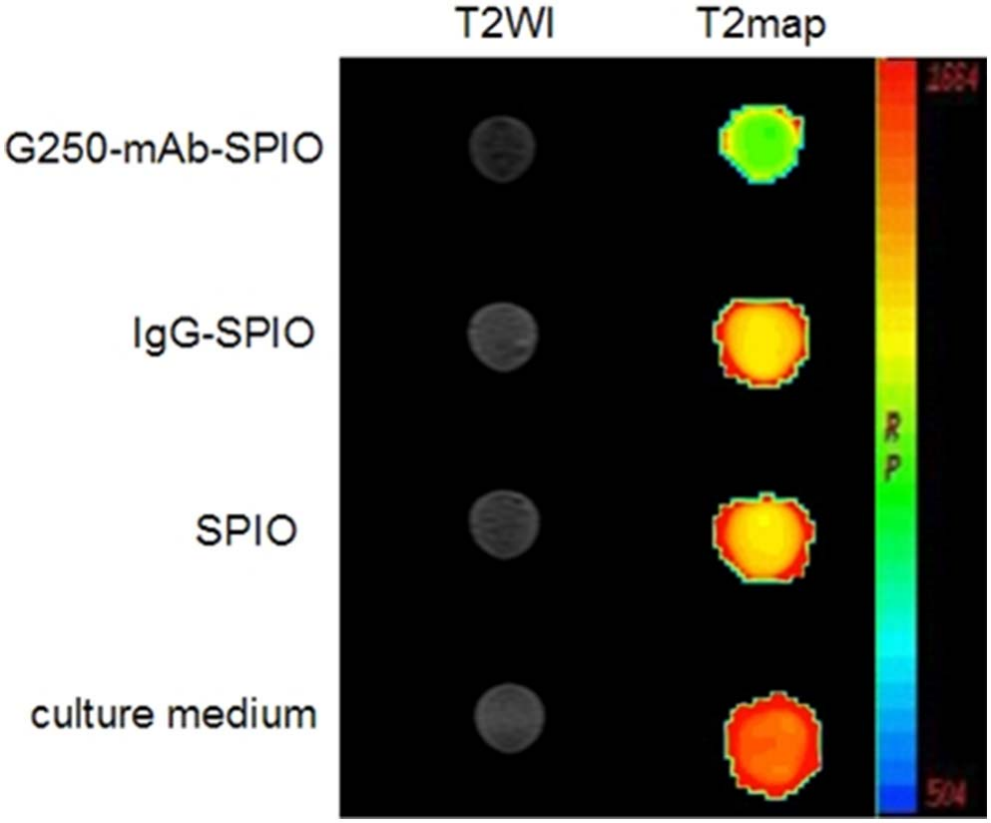
T2-weighted and T2map MR images of 786 -**0 renal carcinoma cells treated with mAb G250-SPIO nanoprobe, IgG-SPIO nanoprobe, SPIO nanoparticles, or culture medium.**

**Figure 8:**
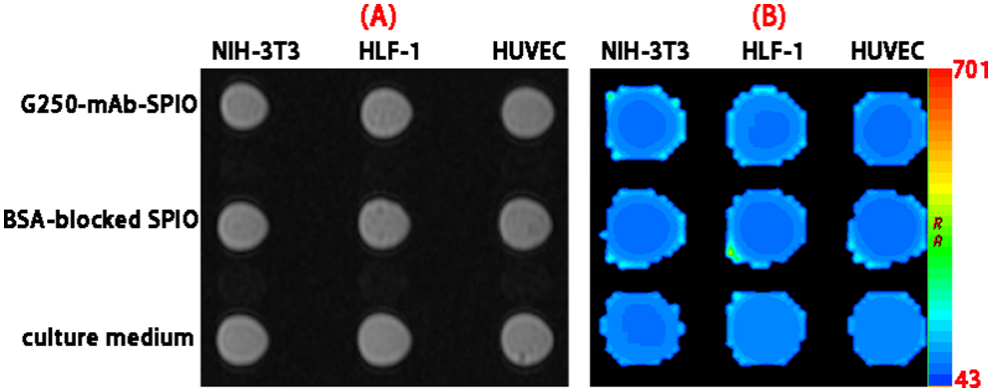
T2-weighted (A) and T2map MR images (B) of NIH-3T3, HLF-1 and HUVEC cells treated with G250-mAb-SPIO nanoprobe, BSA-blocked SPIO nanoparticles, or cell culture medium.

To evaluate the potential application of G250-mAb-SPIO nanoprobe in specific molecular MR imaging, a model for their specific diagnosis of ccRCC by MRI was designed based on the specific binding of G250-mAb to CAIX antigen and 786-0 renal carcinoma cells with over-expression of CAIX antigen. The 786-0 renal carcinoma cells were treated with G250-mAb-SPIO nanoprobe, IgG-SPIO nanoparticles, SPIO nanoparticles, or culture medium for 1 h. After washed with PBS and lysed by typsin, cells were harvested and resuspended in 30% gelatin solution for the following MRI test on a 3.0T scanner using human brain coil. The specific targeting ability of G250-mAb-SPIO nanoprobe for 786-0 renal carcinoma cells was evaluated. T2-weighted MR images of four groups, including cells incubated with G250-mAb-SPIO nanoprobe, IgG-SPIO nanoparticles, SPIO nanoparticles, or culture medium were shown in Figure 7. In the group of G250-mAb-SPIO nanoprobe, a better dark contrast was displayed compared with the other groups of IgG-SPIO nanoparticles, SPIO nanoparticles, or culture medium, indicating the specific cellular binding of G250-mAb-SPIO nanoprobe to G250 receptors on 786-0 renal carcinoma cells. The T2 relaxation times of each group were calculated by function software at a workstation (ADW 4.2) and the values for G250-mAb-SPIO nanoprobe, IgG-SPIO nanoparticles, SPIO nanoparticles, and culture medium were 54.06±2.30, 75.36±4.83, 72.28±3.18, 91.57±7.02 ms, respectively. The T2 relaxation time value of untreated cells came from the nonspecific adsorption of nanoparticles onto the surface of cells. The little difference between IgG-SPIO group and SPIO group indicated that few control antibody (IgG-mAb) was bound with the target cells. The obvious MRI signal from G250-mAb-SPIO nanoprobe is ascribed to the specific targeting of G250-mAb to G250 receptors on 786-0 renal carcinoma cells. To further confirm this mAb G250-based specific targeting, NIH-3T3, HLF-1 and HUVEC cells were introduced as controls and treated with G250 mAb-SPIO nanoprobe, BSA-blocked SPIO nanoparticles and culture medium, respectively. As shown in Figure 8, no significant difference among these groups and the corresponding data could be found in Table 1. These results show good behavior of our fabricated G250-mAb-SPIO nanoprobe as specific molecular MR imaging probe for ccRCC diagnosis.

**Table 1:** T2 relaxation time of NIH-3T3 cells, HLF-1 cells and HUVEC cells treated with G250-mAb-SPIO nanoprobe, BSA-blocked SPIO nanoparticles or culture medium.

	NIH-3T3	HLF-1	HUVEC
G250-mAb-SPIO	83.37±4.20 ms	85.42±3.78 ms	85.09±2.65 ms
BSA-blocked SPIO	82.90±3.09 ms	83.14±2.65 ms	82.02±3.70 ms
culture medium	90.53±5.14 ms	90.94±5.99 ms	91.10±6.39 ms

## Conclusions

In conclusion, we propose a specific G250-mAb-SPIO nanoprobe for molecular MR imaging of ccRCC 786-0 cells. The excellent MRI response and good biocompatiblity of SPIO nanoparticles show their potential as MRI contrast agent. With the help of the specific targeting ability of mAb G250, the fabricated nanoprobe could recognize clear cell renal carcinoma cells sensitively and specifically. This study demonstrates the promise of G250-mAb-SPIO nanoprobe as a molecular magnetic resonance imaging probe for the early diagnositic imaging of ccRCC that overexpresses the G250 receptor. Additionally, this kind of mMRI nanoprobe could be easily conjugated with other imaging materials [34], [35] and antitumor drugs [36], [37] to realize multimodal imaging and the integration of diagnosis and treatment.
